# Visual deterioration in patients with photoreceptor loss after retinal reattachment surgery

**DOI:** 10.1007/s00417-021-05519-z

**Published:** 2022-01-26

**Authors:** Sana Rasool, Megha Kaushik, Rishika Chaudhary, Karen Blachford, Martin Berry, Robert A. H. Scott, Ann Logan, Richard J. Blanch

**Affiliations:** 1 Sandwell & West Birmingham NHS Trust, Birmingham, UK; 2 Department of Ophthalmology, University Hospitals Birmingham NHS Foundation Trust, Birmingham, UK; 3 Neuroscience & Ophthalmology, Institute of Inflammation and Ageing, University of Birmingham, Birmingham, UK; 4 National Institute of Health Research Surgical Reconstruction and Microbiology Centre, University Hospitals Birmingham NHS Foundation Trust, Birmingham, UK; 5 Axolotl Consulting Ltd, Droitwich, WR9 0JS Worcestershire UK; 6 Division of Biomedical Sciences, Warwick Medical School, University of Warwick, CV4 7HL Coventry, UK; 7 Academic Department of Military Surgery & Trauma, Royal Centre for Defence Medicine, Birmingham, UK

**Keywords:** Retinal detachment, Photoreceptors, Apoptosis, Visual field, Visual acuity, OCT

## Abstract

**Purpose:**

Assess the relationship between photoreceptor degeneration and visual function after retinal reattachment surgery (RRS) in a prospective cohort.

**Methods:**

Patients with rhegmatogenous retinal detachment (RRD) were reviewed before and 6 months after vitreoretinal surgery. Optical coherence tomographical thickness of the outer nuclear layer (ONL), outer retinal segment (ORS), retinal pigmented epithelium to ellipsoid zone (RPE-EZ) and external limiting membrane to EZ (ELM-EZ) were recorded 6 months post-operatively. These were compared to best corrected visual acuity (BCVA) and retinal sensitivity (Humphrey visual field).

**Results:**

Thirteen macula-off and 8 macula-on RRD patients were included. The mean ONL thickness was higher after macula-on RRD compared to macula-off RRD (97.70 ± 3.62 μm vs. 73.10 ± 4.98 μm). In all RRD eyes, every 1 μm decrease in ONL thickness correlated with a 0.052 dB decrease and in retinal sensitivity and every 1 μm decrease in ORS thickness was associated with a 0.062 dB reduction in retinal sensitivity. ORS, ELM-EZ and RPE-EZ thickness did not correlate with BCVA post-RRS.

**Conclusion:**

There was greater ONL and ORS thinning following macula-off compared to macula-on RRD. Correlations between ONL and ORS thinning with decreased retinal sensitivity may be explained by RRD-induced photoreceptor death.

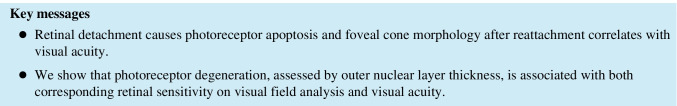

**Supplementary Information:**

The online version contains supplementary material available at 10.1007/s00417-021-05519-z.

## Introduction

There are significant geographical variations in the incidence of rhegmatogenous retinal detachment (RRD) of between 6.3 and 17.9 per 100,000 of population [1]. The visual impairment that ensues after retinal reattachment surgery (RRS) is linked to multiple factors including proliferative vitreoretinopathy, duration of the RRD, pre-operative visual acuity, patient age, post-operative complications such as cataract, and foveal involvement of the detachment [2]. The visual outcomes of macula-off RRD in which the fovea is detached are worse than those of macula-on detachment in which the fovea is still attached, with 28% and 68% achieving 6/12 vision or better at 3 months, respectively [3].

During RRD, separation of the retina from the retinal pigmented epithelium (RPE) leads to outer retinal ischaemia and isolation from the underlying choroidal blood supply [4]. In animal models of RRD, photoreceptor death occurs as early as 12 h peaking around 2–3 days [5].

After reattachment, retinal function is impaired by altered synaptic connectivity in the outer plexiform layer, imperfect photoreceptor outer segment regeneration, failed reconstitution of the RPE cone sheath and scarring within the retina [6]. In human studies, evidence for photoreceptor degeneration after RRS includes thinning of the outer nuclear layer (ONL), reduced cone mosaic density and suppression of genes regulating photoreceptor function [7–11]. Subretinal caspase-8 and -9 levels increase as the area of detachment increases in human RRD and TUNEL-positive nuclei are detected in the ONL 1–7 days after RRD, which are changes that suggest photoreceptors degenerate by apoptosis [12, 13]. Vitreous levels of pro-apoptotic molecules intercellular adhesion molecule-1 (ICAM-1), induced by Fas ligation, and monocyte chemoattractant protein-1 (MCP-1) are also upregulated after RRS [7, 14–16].

Clinically, the mean ONL thickness has been used as a global measure of photoreceptor degeneration in operated and un-operated retinae, as well as foveal cone density [11, 17]. Cone ellipsoid zone thickness changes on OCT have also been associated with visual acuity 12 months post-RRS [17, 18]. In this study, changes in visual function, assessed by best corrected visual acuity (BCVA) and perimetric retinal sensitivity, were studied in parallel with changes in outer retinal thickness, as a function of photoreceptor loss after RRS.

## Methods

This prospective observational cohort study was approved by the National Health Service Research Ethics Service (12/WM/0330) and adhered to the tenets of the Declaration of Helsinki. Before enrolling in this study, informed consent was obtained from all patients [19]. Recruitment criteria included patients undergoing vitreoretinal surgery for primary RRD between January 2015 and July 2017. Exclusion criteria were previous vitreoretinal surgery, age under 10, inability to consent and pregnancy.

A pre-operative baseline ophthalmic examination was recorded. Pre-operative characteristics collected included patient age, gender, coexistent ocular pathology, best-corrected visual acuity (BCVA) in the affected and unaffected eye, macula-on or -off RRD (based on slit-lamp examination), duration of symptoms prior to diagnosis and number of days between diagnosis and RRS.

**Fig. 1:**
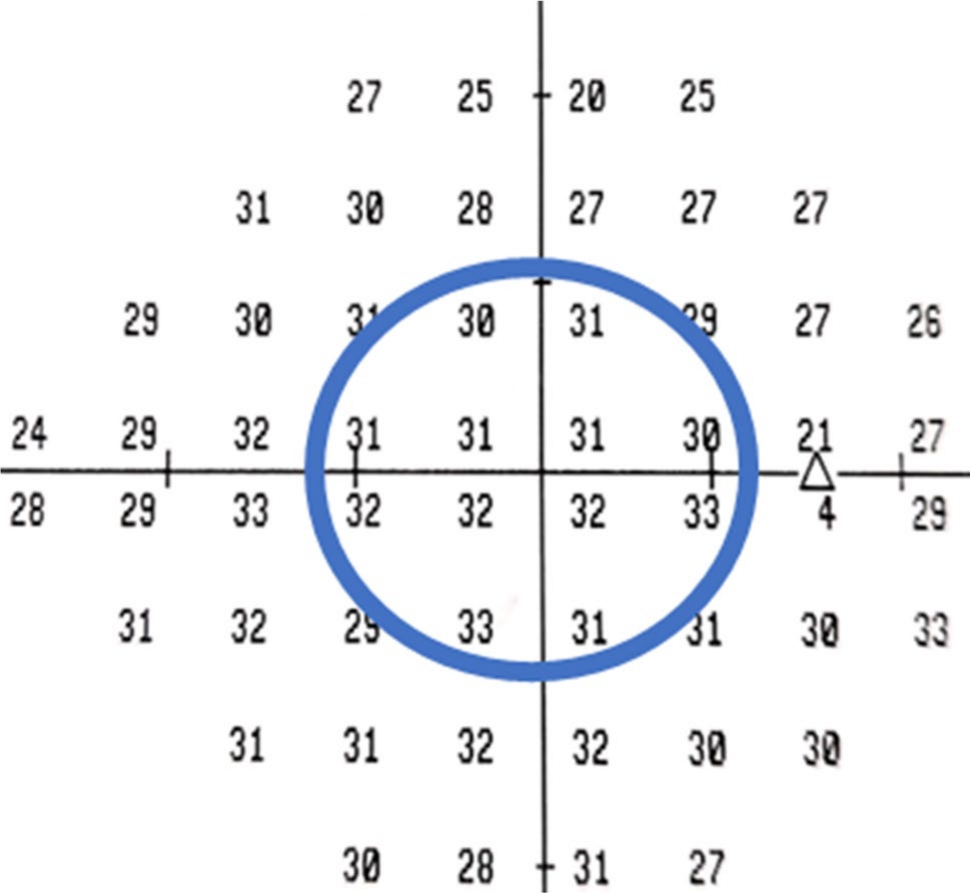
Retinal sensitivity. Derived from the central 12 points around the fovea in a normal eye of an adult male

Six months post-RRS, BCVA, dilated fundus ophthalmoscopy and Humphrey visual fields (HVF) were all performed. HVF with a central 24–2 SITA fast protocol was used (Carl Zeiss Meditec, Dublin, CA, USA). The age-adjusted mean deviation in decibels (dB) was recorded in 12 points centred on the fovea in affected and unaffected eyes (Fig. 1). For the purpose of the study, this sampled central field was termed ‘retinal sensitivity’, although it is accepted that other visual pathway changes may also potentially contribute to this measurement. Mean deviation and pattern standard deviations were also reported.

**Fig. 2:**
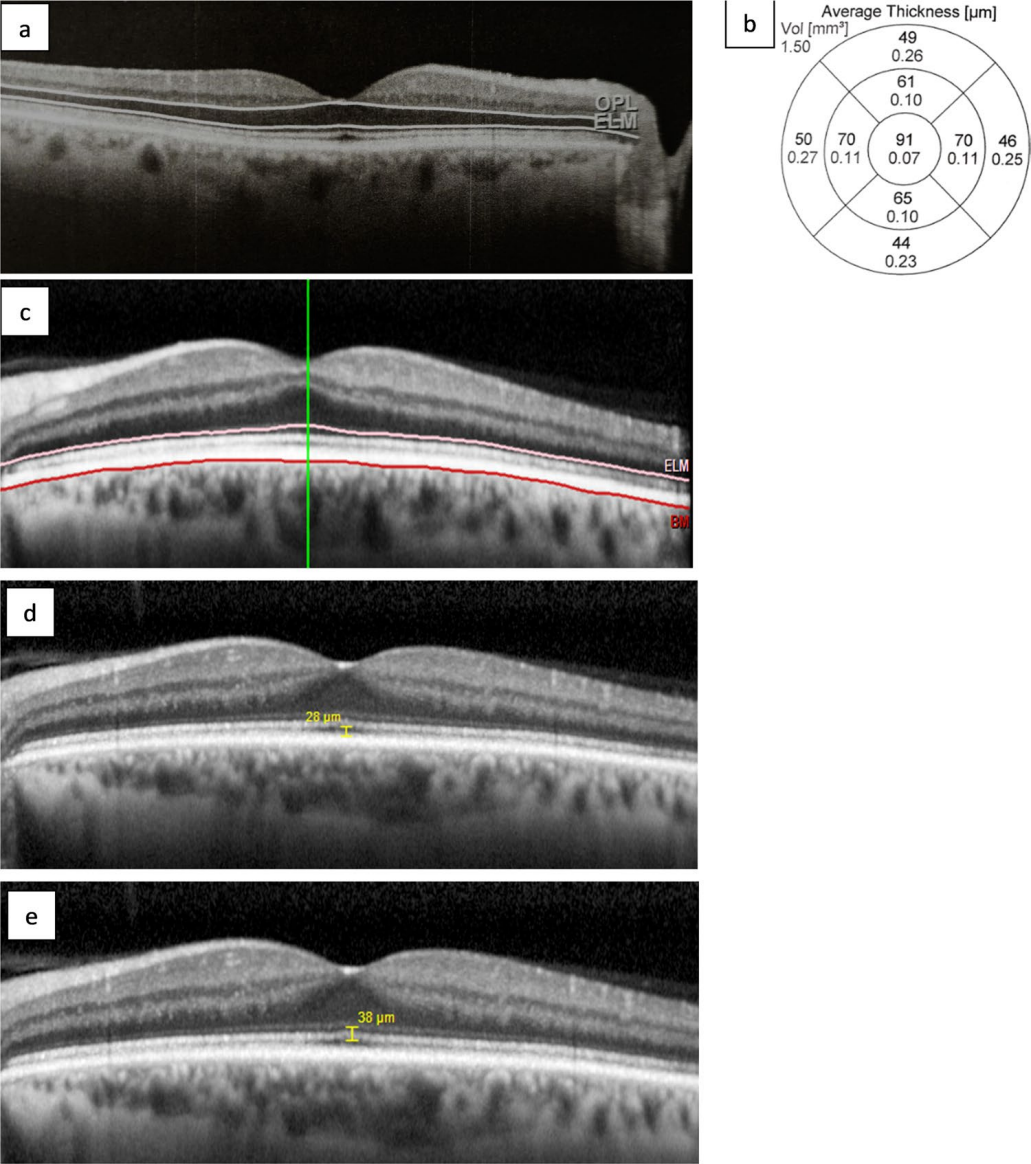
Spectral domain optical coherence tomography. **a** Cross-sectional automated segmentation of the outer nuclear layer (ONL) between the outer plexiform layer (OPL) and external limiting membrane (ELM) in a normal eye of an adult male. **b** ONL thickness in the 9 macula subfields in the corresponding eye. **c** Cross-sectional automated segmentation of the outer retinal segment (ORS) measured from the external limiting membrane (ELM) to Bruch’s membrane (BM). **d** Thickness measured with inbuilt callipers between the inner retinal pigmented epithelium to the outer ellipsoid zone border (RPE-EZ) at the trough of foveal dip. **e** Thickness measured with inbuilt callipers between the outer external limiting membrane border to the outer ellipsoid zone border (ELM-EZ) at the trough of foveal dip

Six months post-RRS, spectral domain optical coherence tomography (SD-OCT Heidelberg version 6.8.3; Heidelberg Engineering Spectralis, Heidelberg, Germany) was performed. OCT volume scans centred on the fovea in affected and unaffected eyes. Automated retinal layer segmentation in the Heidelberg Eye Explorer software was employed and segmentation accuracy was manually verified by ophthalmologists SR and RB (Fig. 2a). If segmentation was inaccurate, new segmentation lines were drawn manually using Heidelberg editing software. ONL thickness was measured in each of the 9 ETDRS (Early Treatment Diabetic Retinopathy Study) macula subfields and the centre point thickness was calculated using a built-in segmentation algorithm by the manufacturer’s proprietary software (Fig. 2b). The outer retinal segment (ORS) thickness was measured from the external limiting membrane (ELM) to Bruch’s membrane in each of the 9 ETDRS macula subfields also via the manufacturer’s inbuilt segmentation software (Fig. 2c). The ONL and ORS thickness were compared to corresponding individual points on the visual field. An Ophthalmologist (SR) used inbuilt callipers to measure the distance between the inner RPE border and outer ellipsoid zone border (RPE-EZ) and the outer ELM border to the outer ellipsoid zone border (ELM-EZ) at a single point at the fovea (Fig. 2d, 2e). Both ELM-EZ and RPE-EZ thickness were compared to BCVA.

Patients were divided into 2 groups based on the retinal attachment status of the macula (macula-on and macula-off) immediately before RRS. Macula status was confirmed by clinical examination before repair and intra-operatively at the start of each vitrectomy. Pre-operative OCT was not undertaken, and thus subclinical submacular fluid in the macula-on group amounting to partial macula detachment cannot be excluded. Statistical comparisons were made between RRD affected eyes and unaffected eyes. For statistical analysis, Snellen visual acuity was converted into the logarithm of the minimum angle of resolution (logMAR). Eyes with a visual acuity of finger count or hand movements were assigned logMAR values of 2.0 and 3.0, respectively [20]. The effect of ONL, ORS, ELM-EZ and RPE-EZ thickness on visual acuity and retinal sensitivity was modelled using generalised estimating equations (GEE) to account for the inclusion of data from both eyes of each subject and multiple corresponding retinal OCT and visual field data points (significant differences between means, including 2-way interaction terms in the initial model and sequential removal of terms, were set at *p* values > 0.05). Sensitivity analysis used a chained equations method to impute five datasets for analysis of the effect of missing and excluded values. Analyses were performed in SPSS version 24 (IBM, Armonk, NY, USA) and means were compared using the Student *t*-test.

## Results

Twenty-four patients were recruited. Three in the macula-off RRD group were excluded from the analysis because post-operative cystoid macula edema distorted the retinal architecture. No patient had post-operative subretinal fluid at the macula. Thirteen with macula-off RRD and 8 with macula-on RRD were included. Two patients did not have a 6 month BCVA recorded, both in the macula-on RRD group. There was male predominance in the macula-off group (Table 1). The mean duration of symptoms for macula-off RRD was 3.9 weeks compared to 6.5 weeks for macula-on RRD patients. The mean duration between presentation and RRS was 12 days for the macula-off RRD group and 8 days for the macula-on RRD group. All patients had 3-port pars plana vitrectomy. Concurrent cataract surgery was not performed in any case. Eleven patients had cataract in the affected eye (8 macula-off RRD), two had no cataract and two were pseudophakic. Cataract status was not documented for 6 patients. There was a significant difference in the mean BCVA between patients with coexistent cataract and those without cataract (logMAR 1.37 versus 0.27, *p* = 0.02). No patients had any relevant systemic or other ocular co-morbidities including high myopia. Patient demographics, OCT and visual field data are summarised in Table 1.

**Table 1:** Patient characteristics and 6-month post-operative optical coherence tomography and Humphrey visual field measurements in the macula-off and macula-on rhegmatogenous retinal detachment (RRD) groups

	**Macula-off RRD**	**Macula-on RRD**
**Age, mean ± *SD* (range)**	69.7 ± 10.2 (51–85)	62.4 ± 7.7 (49–74)
**Sex, *N***		
Male	11	5
Female	2	3
**Best corrected visual acuity, mean ± SD LOGMAR**		
At presentation	1.74 ± 1.04	− 0.1 ± 0.13
6 months post-operative	0.76 ± 0.51	0.42 ± 0.54
**Duration of symptoms, mean ± *SD* weeks (range)**	3.9 ± 3.2 (1–11)	6.5 ± 4.9 (1–12)
**Duration from presentation to surgery, mean ± *SD* days (range)**	12.4 ± 19.3 (0–70)	7.6 ± 20.8 (0–59)
**Central retinal thickness, mean ± *SD* µm (range)**		
Affected eyes	312.73 ± 46.23 (234–381)	321.71 ± 46.99 (252–369)
Unaffected eyes	322.25 ± 89.67 (264– 602)	306.88 ± 36.40 (254–349)
**Outer nuclear layer thickness, mean ± *SD* µm**		
Affected eyes	73.10 ± 4.98	97.70 ± 3.62
Unaffected eyes	93.75 ± 4.99	95 ± 9.81
**Outer retinal segment thickness, mean ± *SD* µm**		
Affected eyes	91.50 ± 8.66	93.00 ± 4.40
Unaffected eyes	91.83 ± 4.61	92.14 ± 3.63
**RPE-EZ, mean ± *SD* µm**		
Affected eyes	21.67 ± 6.27	26.00 ± 9.01
Unaffected eyes	26.75 ± 5.86	24.50 ± 3.25
**ELM-EZ, mean ± *SD* µm**		
Affected eyes	32.89 ± 5.51	40.86 ± 10.17
Unaffected eyes	37.67 ± 5.83	37.12 ± 4.05
**Retinal sensitivity, mean ± *SD* dB**		
Affected eyes	28.3 ± 2.72	30.2 ± 2.27
Unaffected eyes	30.4 ± 4.21	31.3 ± 1.73
**Mean deviation, mean ± *SD* dB**		
Affected eyes	-4.16 ± 2.12	-3.7 ± 3.17
Unaffected eyes	-3.44 ± 9.18	-0.9 ± 2.00
**Pattern standard deviation, mean ± *SD* dB**		
Affected eyes	2.03 ± 0.48	2.88 ± 2.62
Unaffected eyes	2.49 ± 1.58	2.35 ± 1.46

*SD*, standard deviation; *RPE-EZ*, retinal pigmented epithelium–ellipsoid zone; *ELM-EZ*, external limiting membrane–ellipsoid zone

Six months post-RRS, the mean ONL thickness modelled across the 9 EDTRS grid areas was 97.70 ± 3.62 μm after macula-on RRS compared to 73.10 ± 4.98 μm after macula-off RRS (*p* = 0.008).

The mean age in the macula-on RRD group was 62.4 ± 7.7 years and was 69.7 ± 10.2 years in the macula-off RRD group. ONL thickness declined with every year of advancing age by 0.475 µm (0.006–0.945; *p* = 0.047), and there was no evidence that this differed between macula-on RRD eyes, macula-off RRD eyes and unaffected eyes. In the macula-off RRD group, the ONL was 10.7 µm (3.35–18.1) thinner than in the macula-on group.

### Outer retinal thickness and HVF retinal sensitivity

In all affected and unaffected eyes (in both macula-on and -off groups), ONL thickness 6 months after RRS strongly correlated with retinal sensitivity (*p* = 0.001). Eyes affected by RRD had a reduction in retinal sensitivity of 1.874 dB (0.44–3.31) compared to unaffected eyes. Retinal sensitivity was reduced by 0.052 dB (0.021–0.084) per µm reduction in ONL thickness after both macula-off and macula-on RRD (supplementary Figure). The type of RRD (macula-on vs. macula-off) did not predict retinal sensitivity when ONL thickness was included in the model (*p* = 0.619) and similarly age, symptom duration and time between presentation and RRS (all included in the initial GEE model) were not independently associated with retinal sensitivity.

After accounting for missing data (those of patients excluded for cystoid macular edema), there was still an association between ONL thickness and retinal sensitivity. A sensitivity analysis with 5 imputed data sets in macula-on (8 patients) and -off eyes (13 patients) yielded pooled results consistent with the primary analysis, i.e. with every 1 µm decrease in ONL thickness, a reduction in retinal sensitivity of 0.109 dB (0.038–0.180) was recorded. There was also an association between ORS thickness and localised retinal sensitivity (*p* = 0.032), whereby every 1 µm decrease in ORS thickness was associated with a 0.062 dB (0.005–0.119) reduction in retinal sensitivity.

### Outer retinal thickness and BCVA

The mean BCVA for macula-off RRD improved from logMAR 1.74 ± 1.04 pre-operatively to 0.76 ± 0.51 post-operatively (*p* = 0.004), but did not change significantly for macula-on RRD (− 0.1 ± 0.13 to 0.42 ± 0.54, *p* = 0.210).

Visual acuity at 6 months post-RRS was positively associated with ONL thickness in the central EDTRS subfield (*p* = 0.012). With every 1 µm increase in ONL thickness, there was an improvement in logMAR visual acuity of 0.008 (0.002–0.015). However, this relationship was lost when RRD type (macula-on and -off) and eye status (affected and unaffected) covariates were added to the GEE model (*p* = 0.945). After accounting for missing data by analysing pooled results from the 5 imputed datasets, ONL thickness did not correlate with 6 month BCVA (*p* = 0.289). There was no association between ELM-EZ, RPE-EZ or central ORS thickness and BCVA. Age, duration of symptoms and time from presentation to RRS were also not independently associated with BCVA.

### Qualitative OCT analysis

**Fig. 3:**
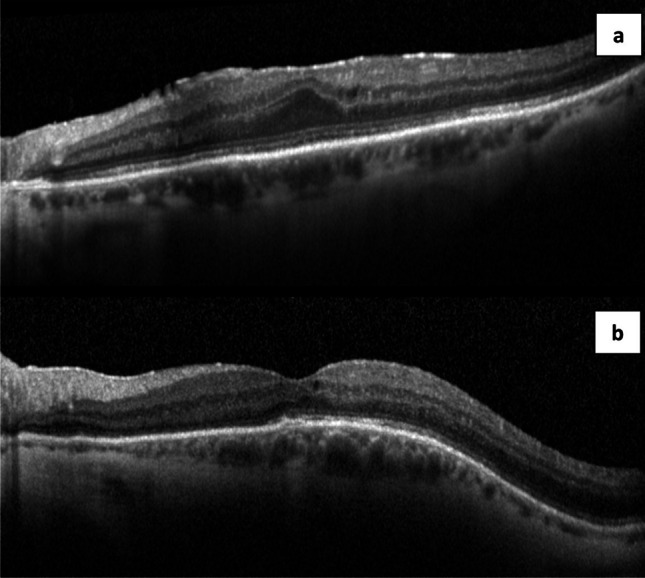
Optical coherence tomography structural appearance 6 months post retinal reattachment surgery. **a** Recovered outer retinal structures post macula-off rhegmatogenous retinal detachment repair. **b** Disrupted outer retinal structures post macula-off rhegmatogenous retinal detachment repair

The macula-on RRD patients all retained normal outer retinal structure at the fovea. Of the macula-off RRD patients, 3 showed recovered outer retinal structures at the fovea (representative image in Fig. 3a), whilst the remainder showed persistent disruption of the outer retina (representative image in Fig. 3b).

## Discussion

We investigated the relationship between outer retinal thickness and visual acuity and perimetric retinal sensitivity. Central ONL thickness strongly associated with retinal sensitivity and less strongly with BCVA.

The association between ONL thickness and BCVA was lost when macula status of the detachment was added to the GEE model, consistent with macula-off RRD reducing both ONL thickness and visual acuity and suggesting that the relationship between ONL thickness and BCVA is mediated by the occurrence of a macula-off RRD.

The associations between ONL thickness and retinal sensitivity and visual acuity support the hypothesis that photoreceptor apoptosis after macula-off retinal detachment contributes to reduced visual function. Because we used clinical examination to distinguish macula-off and -on RRD, it is possible that some macula-on RRD patients may have had subclinical subretinal fluid in the macula, limiting our ability to make distinctions between the relationships in macula-on as opposed to macula-off RRD. A recent smaller study after macula-off RRD repair found similar changes in photoreceptor morphology measured by adaptive optics OCT and retinal sensitivity [21]. Some retinal detachment studies have also shown an association between visual acuity and central ONL thickness [17, 22], photoreceptor morphology and cone packing density [8, 9, 11, 18]. However, as reflected in our study, this relationship has previously been found to be tenuous. For example, in early glaucoma, visual field defects correlate more with contrast acuity than visual acuity and, in age-related macular degeneration, visual field defects and contrast sensitivity are associated with navigational impairment at least as much as visual acuity [23–25], suggesting that visual acuity does not completely characterise the functional effects of photoreceptor loss after RRD.

Whilst significant recovery in photoreceptor morphology could be expected to occur by 6 to 12 months after RRS, inner segment thickness may continue to increase beyond the 12-month post-operative period [18]. Thus, in this study, the effect of photoreceptor survival on visual acuity and the central visual field after RRD may have been masked by delayed recovery of outer or inner segment morphology. The association between ORS thickness and localised retinal sensitivity supports the additional importance of recovery of the photoreceptor inner and outer segment on localised retinal function, whilst the lack of association between RPE-EZ and ELM-EZ and visual acuity suggests that other factors, such as the ultrastructural relationship between the cone outer segments and RPE cone sheath, may also mediate reduced visual acuity. Foveal ELM-EZ and RPE-EZ thickness may be increased by distorted retinal architecture at the centre of the fovea or may be decreased by photoreceptor damage or loss, both of which could reduce visual acuity, meaning that no overall effect of changes in ELM-EZ and RPE-EZ distances may be seen when both effects are present. In addition, another larger study found an association between RPE-EZ and visual acuity at multiple time-points after RRS, suggesting that the present study may be underpowered with respect to this outcome [26]. ORS automated measurements averaged across each of the 9 ETDRS fields would be expected to be less affected by focal disturbances to retinal anatomy and measurement error. Additional variation in visual function may derive from cataract development, which probably explains the difference in retinal sensitivity between affected and unaffected eyes.

The duration of symptoms and time to RRS were not independently associated at 6 months post-RRS with either BCVA, retinal sensitivity, or ONL thickness. Whilst it was expected that a reduction in ONL thickness would occur between macula-off RRD onset and RRS as photoreceptor apoptosis proceeds until reattachment, we did not find an association. This may relate to inconsistent reporting by patients of the exact dates of symptom onset and duration or an inconsistent relationship between onset of symptoms and onset of RRD. In addition, human and animal studies suggest that photoreceptor apoptosis peaks 2–3 days after RRD. However, the median time from presentation to repair of macula-off RRD was 5 days (interquartile range 5–8.5), meaning that most of the macula-off detachments were repaired after peak apoptosis [13]. It is also possible that the study was underpowered with respect to this outcome.

The relationship between age and photoreceptor density has previously been reported [27]. However, we did not find an association between age and visual acuity or retinal sensitivity, which may be attributed to masking confounders discussed above such as follow-up interval, coexistent disease, e.g. cataract, and small sample size especially with respect to incomplete post-operative BCVA data. As the mean BCVA was worse in patients with coexistent cataract, this may have masked some of the relationship between BCVA and retinal morphology.

We have shown that central retinal sensitivity, as assessed by Humphrey perimetry and visual acuity, correlate with ONL thickness after macula-off RRS, providing evidence that photoreceptor degeneration after RRS contributes to reduced visual function.

## Supplementary Information

Below is the link to the electronic supplementary material.

Supplementary file1 (PDF 116 KB)
