# Supplementary material for: Visual deterioration in patients with photoreceptor loss after retinal reattachment surgery
Source: Graefes Arch Clin Exp Ophthalmol. 2022 Jan 26;260(7):2141–7. doi: 10.1007/s00417-021-05519-z (PMC9203401; doi:10.1007/s00417-021-05519-z)

VISUAL DETERIORATION IN PATIENTS WITH PHOTORECEPTOR LOSS AFTER RETINAL REATTACHMENT SURGERY

Rasool S, Kaushik M, Chaudhary R, Blachford K, Berry M, Scott RAH, Logan A, Blanch RJ.

Supplementary Figure 1. Modelled relationship between outer nuclear layer thickness (ONL; μm) and Humphrey Visual Field sensitvity (HVF; dB).

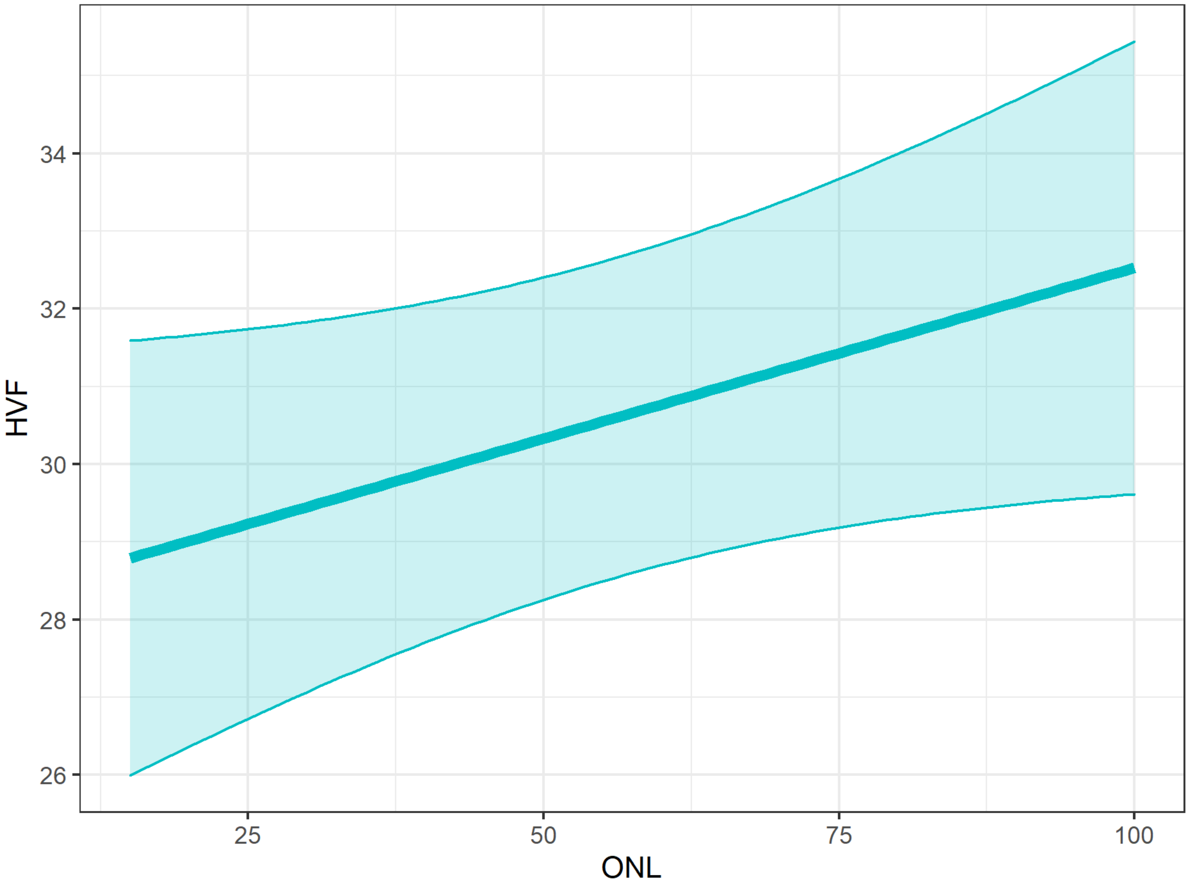

Supplement: Supplementary file 1 — Supplementary file1 (PDF 116 KB) [file 417_2021_5519_MOESM1_ESM.pdf]
